# SLC26A4-AS1 Agrava a Hipertrofia Cardíaca Induzida por AngII Aumentando a Expressão de SLC26A4

**DOI:** 10.36660/abc.20210933

**Published:** 2023-04-12

**Authors:** Xiaoliang Han, Chao Li, Qinjiong Ji, Ling Zhang, Xiaofei Xie, Huijuan Shang, Hong Ye

**Affiliations:** 1 Departamento de Cardiologia Instituto de Controle de Tuberculose de Anhui Hefei Anhui China Departamento de Cardiologia, Anhui Provincial Chest Hospital, (Instituto de Controle de Tuberculose de Anhui), Hefei, Anhui – China; 2 Departamento de Cardiologia Hospital Hefei Medical University of Anhui Hefei Anhui China Departamento de Cardiologia, the Second People’s Hospital of Hefei (Hospital Hefei afiliado à Medical University of Anhui), Hefei, Anhui – China

**Keywords:** Cardiomegalia, RNA, RNA Longo não Codificante

## Abstract

**Fundamento:**

Foi relatado que o RNA 1 antisenso 1 (SLC26A4-AS1) do membro 4 da família de transportadores de soluto 26 está altamente relacionado à hipertrofia cardíaca.

**Objetivo:**

Esta pesquisa visa investigar o papel e o mecanismo específicos de SLC26A4-AS1 na hipertrofia cardíaca, fornecendo um novo marcador para o tratamento da hipertrofia cardíaca.

**Métodos:**

Angiotensina II (AngII) foi infundida em cardiomiócitos ventriculares (NMVCs) de camundongos neonatos para induzir hipertrofia cardíaca. A expressão gênica foi detectada por PCR quantitativo em tempo real (RT-qPCR). Os níveis de proteína foram avaliados por western blot. Ensaios funcionais analisaram o papel de SLC26A4-AS1. O mecanismo de SLC26A4-AS1 foi avaliado por imunoprecipitação de proteína de ligação a RNA (RIP), pull-down de RNA e ensaios de luciferase repórter. O valor de p < 0,05 foi identificado como significância estatística. O teste t de Student avaliou a comparação dos dois grupos. A diferença entre os diferentes grupos foi analisada por análise de variância (ANOVA) de uma via.

**Resultados:**

SLC26A4-AS1 é regulado para cima em NMVCs tratados com AngII e promove hipertrofia cardíaca induzida por AngII. SLC26A4-AS1 regula o membro 4 da família de transportadores de soluto 26 (SLC26A4) por meio do funcionamento como um RNA endógeno competitivo (ceRNA) para modular o microRNA (miR)-301a-3p e o miR-301b-3p em NMVCs. SLC26A4-AS1 promove hipertrofia cardíaca induzida por AngII via regulação para cima de SLC26A4 ou absorção de miR-301a-3p/miR-301b-3p.

**Conclusão:**

SLC26A4-AS1 agrava a hipertrofia cardíaca induzida por AngII via absorção de miR-301a-3p ou miR-301b-3p para aumentar a expressão de SLC26A4.

## Introdução

A hipertrofia cardíaca é uma resposta adaptativa para melhorar a pós-carga cardíaca para sustentar a função cardíaca sistólica nos estágios iniciais.^1^ Apesar disso, a hipertrofia miocárdica contínua é uma doença cardíaca típica induzida pelo aumento da carga de trabalho do coração acompanhada de remodelação cardíaca mal adaptativa, levando à estenose aórtica e cardiomiopatia dilatada.^2 , 3^ Paredes ventriculares excessivamente espessas e cardiomiócitos são os padrões primários de hipertrofia cardíaca.^2 , 3^ Nos últimos anos, a hipertrofia cardíaca tornou-se um problema urgente que pode levar a uma incidência crescente de insuficiência cardíaca e morte súbita.^3^

As características marcantes da hipertrofia cardíaca patológica são a elevação de genes fetais como o peptídeo natriurético cerebral (BNP), peptídeo natriurético atrial (ANP) e a cadeia pesada de beta-miosina (β-MHC).^4^ Estudos recentes provaram que muitos fatores, incluindo fatores de crescimento específicos, hormônios peptídicos e microRNAs (miRNAs), são responsáveis pela hipertrofia cardíaca,^5^ mas o mecanismo molecular exato subjacente à progressão da hipertrofia cardíaca permanece obscuro.

Os RNAs longos não codificantes (lncRNAs) participam de processos patológicos por meio do controle da expressão gênica nos níveis epigenético, transcricional e pós-transcricional.^6^ Evidências crescentes revelaram os papéis vitais dos lncRNAs no desenvolvimento de doenças cardiovasculares, incluindo a hipertrofia cardíaca.^7^ Por exemplo, o lncRNA SNHG14 induzido por SP1 promove hipertrofia cardíaca via rede miR-322-5p/miR-384-5p/PCDH17 em cardiomiócitos induzidos por angiotensina II (AngII).^8^ O silenciamento de LncRNA MIAT restringe a hipertrofia cardíaca induzida por AngII via eixo de RNA endógeno concorrente miR-93/TLR4 (ceRNA).^9^ O membro 4 da família LncRNA transportadora de soluto 26 RNA antisenso 1 (SLC26A4-AS1) foi relatada como altamente associada com hipertrofia cardíaca. ^10^ No entanto, o efeito e o mecanismo regulador do SLC26A4-AS1 na hipertrofia cardíaca permanecem vagos.

Os miRNAs são outro tipo de RNA não codificante com um comprimento de cerca de 22 nucleotídeos.^11^ Os miRNAs geralmente exercem funções celulares por meio do direcionamento de RNAs mensageiros a jusante (mRNAs).^12^ Numerosos miRNAs têm sido apontados como reguladores essenciais na progressão da hipertrofia cardíaca.^13^ Por exemplo, o miR-17-5p facilita a hipertrofia cardíaca induzida por AngII por meio da inibição da expressão de Mfn2 e ativação do eixo PI3K/AKT/mTOR para suprimir a autofagia.^14^ O MiR-29a melhora a hipertrofia cardíaca induzida pelo cloridrato de isoproterenol ao reprimir a expressão do PPARδ.^15^ Considerando o papel vital dos miRNAs na hipertrofia cardíaca, tentamos investigar o mecanismo regulador do SLC26A4-AS1 focando nos miRNAs que interagem com ele.

No estudo atual, o efeito de SLC26A4-AS1 no nível de BNP, ANP e β-MHC foi explorado para determinar a função de SLC26A4-AS1 na hipertrofia cardíaca induzida por AngII. O mecanismo regulador de SLC26A4-AS1 foi investigado através do eixo ceRNA. Nosso estudo pode oferecer uma nova visão sobre os mecanismos fundamentais do lncRNA na hipertrofia cardíaca, fornecendo um novo alvo para o tratamento da hipertrofia cardíaca.

## Métodos

### Cultura celular e tratamento

Os cardiomiócitos foram isolados dos camundongos recém-nascidos. O comitê de ética do nosso Hospital aprovou experimentos com animais. Para induzir a hipertrofia dos cardiomiócitos, os corações extraídos foram partidos em pedaços e tratados com tripsina 0,25% (1/400, Beyotime, Shanghai, China), seguido de incubação em meio Eagle modificado por Dulbecco/F-12 (500mL, Thermo Fisher Scientific, Rockford, IL, EUA) com 10% FBS (1/10, Invitrogen, Carlsbad, CA, EUA). Após a confluência celular atingir 80%, as células nas placas foram transferidas para placas de cultura e inoculadas com AngII (150 nM, Sigma-Aldrich, St. Louis, MO, EUA) com a concentração de 150 nM para 1, 6, 12 e 24 h para estimular fenótipos hipertróficos de cardiomiócitos, estabelecendo o modelo celular de hipertrofia cardíaca.

### Transfecção celular

Foram todos adquiridos da GenePharma Co., Ltd. (Shanghai, China) grampos curtos de RNA (shRNAs) contra SLC26A4-AS1 (sh-SLC26A4-AS1#1/2, 3ul/ug, GenePharma Co., Ltd., Xangai, China), shRNAs contra o membro 4 da família 26 de transportador de soluto (SLC26A4) (sh -SLC26A4#1/2, 3ul/ug, GenePharma Co., Ltd.), controle negativo de shRNA (sh-NC, 3ul/ug, GenePharma Co., Ltd.), pcDNA3.1-SLC26A4 (3ul/ug, GenePharma Co., Ltd.), vetor vazio pcDNA3.1 (pcDNA3.1, 3ul/ug, GenePharma Co., Ltd.), microRNA (miR)-301a-3p mimético (3ul/ug, GenePharma Co., Ltd. ), miméticos de miR-301b-3p (3ul/ug, GenePharma Co., Ltd.), controle negativo de miméticos de miRNA (miméticos de NC, 3ul/ug, GenePharma Co., Ltd.), inibidor de miR-301a-3p (3ul /ug, GenePharma Co., Ltd.), inibidor de miR-301b-3p (3ul/ug, GenePharma Co., Ltd.) e controle negativo do inibidor de miRNA (inibidor de NC, 3ul/ug, GenePharma Co., Ltd.). Todas as transfecções celulares foram aplicadas usando Lipofectamine 3000 (1,5ug/ml, Invitrogen), seguido pelo tratamento de 150 nM AngII por 24 h.

### PCR quantitativo em tempo real (RT-qPCR)

O RNA total das células foi extraído pelo reagente TRIzol (1mL, Invitrogen). A concentração total de RNA foi determinada medindo o valor de absorção a 260 nm com um microespectrofotômetro. O RNA (1μl) foi transcrito reversamente para compor o DNA complementar usando a transcriptase reversa M-MLV (10000u, Promega, Madison, WI, EUA). Em seguida, a qPCR foi realizada com o kit SYBR Premix Ex Taq™ II (Takara, Japão). A expressão de miRNA foi normalizada para U6, e a de lncRNA e mRNA foi normalizada para gliceraldeído-3-fosfato desidrogenase (GAPDH), que foi baseado no método 2−ΔΔCt. A análise por RT-qPCR foi realizada conforme descrito anteriormente.^16^ Três ensaios independentes foram conduzidos. A sequência do iniciador para SLC26A4-AS1 e GAPDH é mostrada na Tabela 1 .

**Tabela 1:** – Sequência de iniciadores para SLC26A4-AS1 e GAPDH

Genes	Sequência
SLC26A4-AS1	F:TGCTGTTGCTGGAAAGCGAG R: TATTCCTTCCCGCGTGTCCT
GAPDH	F: GACAGTCAGCCGCATCTTCT R:GCGCCCAATACGACCAAATC

*F: sequência direta; R: sequência reversa.*

### Western blot

Foi usado tampão de lise RIPA (100mL, Thermo Fisher Scientific) para extrair proteínas totais, e a concentração de proteína foi verificada usando um kit de ensaio de proteína BCA (Abcam, EUA). Após o tratamento de eletroforese em gel de poliacrilamida e dodecil sulfato de sódio (SDS-PAGE, 1/10, Invitrogen), as proteínas foram transferidas para membrana de PVDF (Millipore, EUA) e bloqueadas em leite desengordurado a 5%. Posteriormente, a membrana foi cultivada com anticorpos primários contra ANP (1µg/ml, ab237632, Abcam, Cambridge, MA, USA), BNP (1µg/ml, ab92500, Abcam), β-MHC (1/2500, ab55152, Abcam), SLC26A4 (0,25 µg/μl, 20889-1-AP, Proteintech, Chicago, EUA) e GAPDH (1/2500, ab8245, Abcam) a 4°C durante a noite. Após a lavagem, a membrana foi cultivada com anticorpo secundário conjugado com HRP (1/2500, Abcam) por 1 h. O sinal foi medido usando o substrato de western blotting de quimioluminescência melhorada. Todos os experimentos independentes foram repetidos três vezes.

### Coloração de imunofluorescência (IF) e ensaio de área de superfície celular

As células foram lavadas e fixadas e bloqueadas com soro de cabra normal contendo 1% de BSA (1/100, Sigma-Aldrich). Posteriormente, as células foram cultivadas com anti-α-actinina (1/100, Sigma-Aldrich) a 4°C por uma noite inteira e foram incubadas com um anticorpo secundário por 1 h. DAPI (4’,6-diamidino-2-fenilindole, 1/2000, Abcam) foi aplicado para corar os núcleos, e um microscópio de fluorescência foi utilizado para capturar as imagens e avaliar a área de superfície celular. Três ensaios independentes foram conduzidos.

### Fracionamento subcelular

Um kit PARIS (Thermo Scientific, EUA) foi usado para isolar as frações citoplasmática e nuclear de cardiomiócitos ventriculares (NMVCs) de camundongos neonatos. A expressão de GAPDH, U6 ou SLC26A4-AS1 no núcleo e no citoplasma foi avaliada por análise de RT-qPCR. Três ensaios independentes foram conduzidos.

### Hibridação fluorescente in situ (FISH)

As sondas SLC26A4-AS1 marcadas com Alexa Fluor 555 (50 ng, RiboBio, Guangzhou, China) foram sintetizadas pela RiboBio (Guangzhou, China). Os experimentos FISH foram implementados com um kit FISH (RiboBio). As células foram semeadas em lâminas por 24 horas de incubação. Após fixação e permeabilização, as células foram cultivadas com sondas SLC26A4-AS1 a 37°C durante a noite. O DAPI foi utilizado para corar os núcleos das células e as imagens foram observadas em microscópio de fluorescência. Bio-repetições foram realizadas em triplicado.

### Imunoprecipitação de proteínas de ligação ao RNA (RIP)

Com base nas instruções, o kit Magna RIP de imunoprecipitação de proteína de ligação a RNA (Millipore) e o anticorpo Ago2 (1/30, Abcam) foram usados para realizar ensaios RIP. Os RNAs coprecipitados foram analisados via RT-qPCR. Três ensaios independentes foram conduzidos.

### Ensaio pull-down de RNA

Os NMVCs foram transfectados com uma sonda SLC26A4-AS1 biotinilada por 48 h. Em seguida, as células coletadas foram cultivadas com esferas magnéticas de estreptavidina M-280 (10 mg/ml, Invitrogen). Os RNAs ligados foram avaliados via RT-qPCR. Três ensaios independentes foram conduzidos.

### Ensaio da luciferase repórter

A sequência de SLC26A4-AS1 ou SLC26A4 3’UTR contendo o local de ligação previsto com miR-301a-3p e miR-301b-3p foi clonada várias vezes no vetor de luciferase dupla pmirGLO (1 µg, Promega), bem como o vetor putativo e sequências mutantes. As células (3 × 10^4^/poço) foram cultivadas em 24 placas poço para incubação. As células foram então transfectadas com esses plasmídeos repórteres e miméticos de miR-301a-3p ou miméticos de miR-301b-3p, respectivamente. Após 48 h de transfecção usando o reagente Lipofectamine 3000, um kit Dual-Luciferase Reporter Assay (Promega) foi aplicado para medir a atividade relativa da luciferase. Três ensaios independentes foram conduzidos.

### Análise estatística

Todos os experimentos foram em triplicata e todos os experimentos independentes foram repetidos três vezes. O tamanho da amostra neste estudo foi definido pelo método de amostragem aleatória e calculado pela fórmula n=2σ2/δ2 f(α, β), onde δ é a diferença mínima, σ é o desvio padrão total, α é o nível de inspeção (= 0,05), e β é a taxa de erro tipo II (=0,10). Os dados foram apresentados como a média ± desvio padrão para 3 ensaios independentes. O software GraphPad Prism versão 5.0 foi aplicado para analisar os dados. A normalidade dos dados foi verificada por meio do teste de Shapiro-Wilk, que mostrou que todos os dados seguiram a distribuição normal. A significância estatística entre os grupos foi investigada pelo teste t de Student não pareado ou análise de variância unidirecional, seguido de um teste post hoc (Tukey e Dunnett). Além disso, um valor de p menor que 0,05 foi identificado como estatisticamente significativo.

## Resultados

### SLC26A4-AS1 é regulado para cima na hipertrofia de cardiomiócitos induzida por AngII

**Figura 1:**
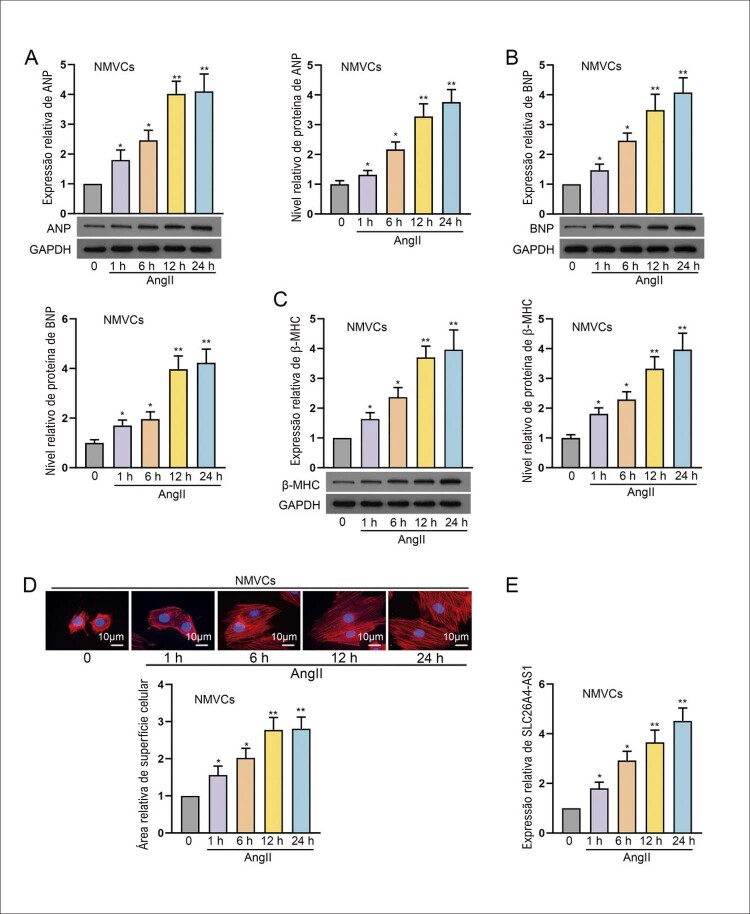
– SLC26A4-AS1 é regulado para cima na hipertrofia cardíaca induzida por AngII. (A-C) Em NMVCs tratados com 150 nM AngII por 1, 6, 12 e 24 h, a expressão de biomarcadores hipertróficos (ANP, BNP e β-MHC) foi avaliada usando RT-qPCR, e o nível de proteína desses biomarcadores foi avaliada por western blot, seguida de quantificação por western blot. *P<0,05: AngII (1 h) vs AngII (0 h). *P<0,05: AngII (6 h) vs AngII (0 h). **P<0,01: AngII (12 h) vs AngII (0 h). **P<0,01: AngII (24 h) vs AngII (0 h). O número de tamanho da amostra (n) = 3. (D) A área de superfície celular em NMVCs tratadas com 150 nM AngII por 1, 6, 12 e 24 h foi avaliada usando coloração IF. Barra de escala: 10 μm. *P<0,05: AngII (1 h) vs AngII (0 h). *P<0,05: AngII (6 h) vs AngII (0 h). **P<0,01: AngII (12 h) vs AngII (0 h). **P<0,01: AngII (24 h) vs AngII (0 h). n = 3. (E) A expressão de SLC26A4-AS1 em NMVCs tratados com 150 nM AngII por 1, 6, 12 e 24 h foi detectada usando RT-qPCR. *P<0,05: AngII (1 h) vs AngII (0 h). *P<0,05: AngII (6 h) vs AngII (0 h). **P<0,01: AngII (12 h) vs AngII (0 h). **P<0,01: AngII (24 h) vs AngII (0 h). n = 3.

Para avaliar o papel específico de SLC26A4-AS1 na hipertrofia cardíaca, primeiro tratamos NMVCs com AngII para induzir fenótipos hipertróficos de cardiomiócitos. Os níveis de biomarcadores hipertróficos (ANP, BNP e β-MHC) em NMVCs tratados com AngII aumentaram gradualmente ao longo do tempo ( Figura 1A-1C ). A coloração mostrou que a área de superfície dos NMVCs tratados com AngII aumentou gradualmente ao longo do tempo ( Figura 1D ). Além disso, uma regulação para cima significativa de SLC26A4-AS1 em NMVCs também foi observada como dependente do tempo após o tratamento com AngII ( Figura 1E ). Em uma palavra, SLC26A4-AS1 é regulado para cima na hipertrofia de cardiomiócitos induzida por AngII.

### SLC26A4-AS1 facilita a hipertrofia cardíaca induzida por AngII

**Figura 2:**
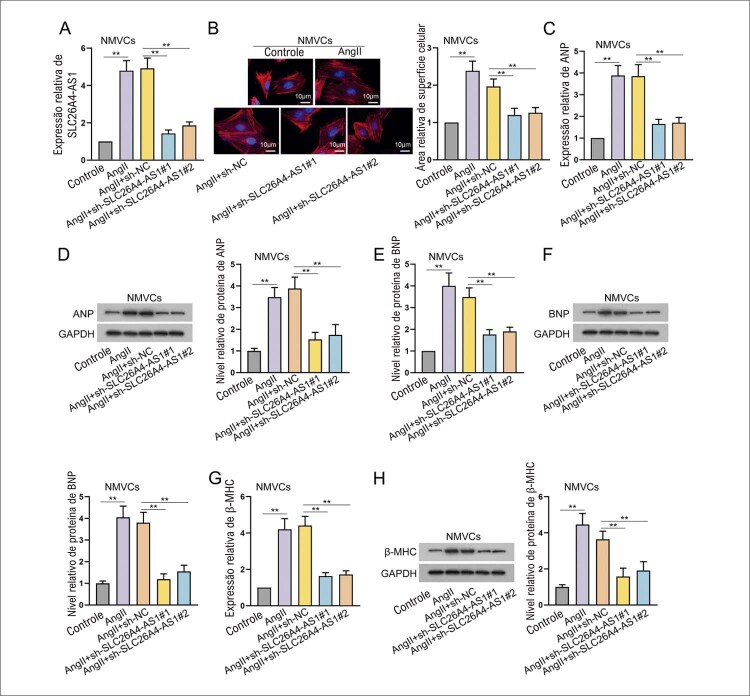
– O silenciamento de SLC26A4-AS1 suprime a hipertrofia cardíaca induzida por AngII. (A) NMVCs foram tratados com sh-SLC26A4-AS1#1/2 ou sh-NC após o tratamento de AngII por 24 h. **P<0,01: AngII vs Controle. **P<0,01: AngII+sh-SLC26A4-AS1#1/2 vs AngII+sh-NC. n = 3. (B) A área da superfície celular foi detectada em NMVCs tratados com AngII após o silêncio de SLC26A4-AS1 usando coloração IF. Barra de escala: 10 μm. **P<0,01: AngII vs Controle. **P<0,01: AngII+sh-SLC26A4-AS1#1/2 vs AngII+sh-NC. n = 3. (C-H) Após o silenciamento de SLC26A4-AS1, a expressão de biomarcadores hipertróficos em NMVCs tratados com AngII foi medida usando RT-qPCR, e o nível desses biomarcadores foi avaliado usando western blot, seguido pela quantificação de western blot. **P<0,01: AngII vs Controle. **P<0,01: AngII+sh-SLC26A4-AS1#1/2 vs AngII+sh-NC. n = 3.

Além disso, a análise de RT-qPCR verificou a transfecção bem-sucedida de plasmídeos sh-SLC26A4-AS1#1/2 em NMVCs induzidos por AngII ( Figura 2A ). A área de superfície celular aumentada em NMVCs causada por AngII foi atenuada pelo silenciamento de SLC26A4-AS1 ( Figura 2B ). Enquanto isso, a expressão regulada para cima e o nível de proteína de ANP, BNP e β-MHC induzidos por AngII foram revertidos após o silenciamento de SLC26A4-AS1 ( Figura 2C-2H ). Esses resultados revelaram que SLC26A4-AS1 promove hipertrofia cardíaca induzida por AngII in vitro.

### SLC26A4-AS1 interage com SLC26A4, e SLC26A4 promove hipertrofia cardíaca induzida por AngII

**Figura 3:**
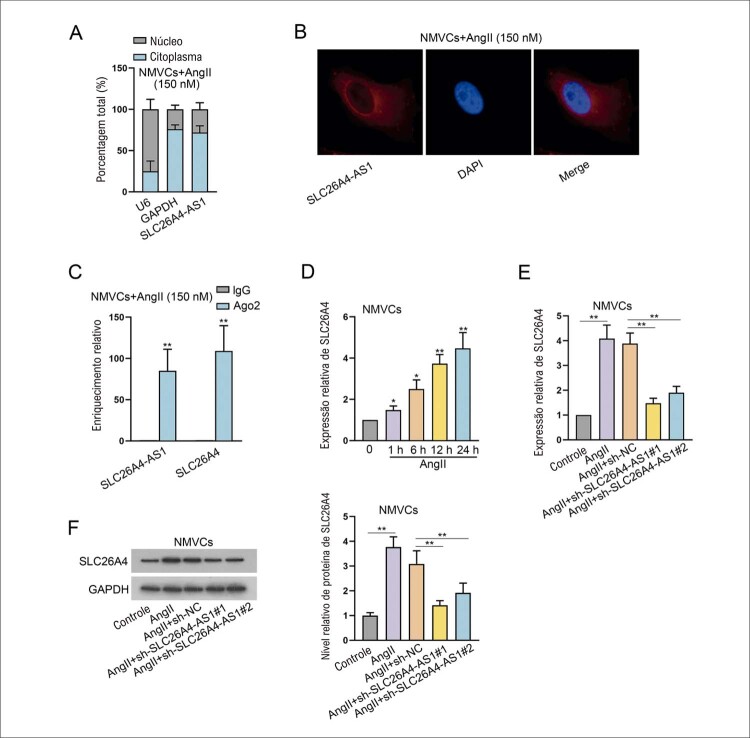
– SLC26A4-AS1 funciona como um ceRNA para regular SLC26A4 na hipertrofia cardíaca induzida por AngII. (A-B) Fracionamento subcelular e ensaios de FISH foram realizados para determinar a localização de SLC26A4-AS1 em NMVCs induzidos por AngII. n = 3. (C) Ensaios RIP detectaram o enriquecimento de SLC26A4-AS1 e SLC26A4. **P<0,01: Ago2 vs IgG. n = 3. (D) A expressão de SLC26A4 em NMVCs tratados com 150 nM AngII por 1, 6, 12 e 24 h foi avaliada usando RT-qPCR. *P<0,05: AngII (1 h) vs AngII (0 h). *P<0,05: AngII (6 h) vs AngII (0 h). **P<0,01: AngII (12 h) vs AngII (0 h). **P<0,01: AngII (24 h) vs AngII (0 h). n = 3. (E-F) O nível de expressão e o nível de proteína de SLC26A4 em NMVCs tratados com AngII foram avaliados usando RT-qPCR e western blot, respectivamente, após regulação negativa de SLC26A4-AS1, seguido pela quantificação de western blot. **P<0,01: AngII vs Controle. **P<0,01: AngII+sh-SLC26A4-AS1#1/2 vs AngII+sh-NC. n = 3.

O fracionamento subcelular e os ensaios de FISH foram implementados pela primeira vez para estudar o mecanismo potencial de SLC26A4-AS1 na hipertrofia cardíaca induzida por AngII. Descobrimos que SLC26A4-AS1 estava preferencialmente localizado no citoplasma celular, sugerindo o papel pós-transcricional de SLC26A4-AS1 em NMVCs tratados com AngII ( Figura 3A-3B ). Foi relatado que os lncRNAs podem regular seus genes próximos para exercer funções no desenvolvimento de doenças.^17^ Conforme mostrado na Figura Suplementar S3A , o banco de dados UCSC (http://genome.ucsc.edu/) previu que SLC26A4 era um gene próximo de SLC26A4-AS1. Portanto, conjecturamos que SLC26A4-AS1 pode regular SLC26A4 para afetar a hipertrofia cardíaca induzida por AngII. Em seguida, ensaios Ago2-RIP foram realizados para que Ago2 seja o componente central do complexo de silenciamento induzido por RNA. SLC26A4-AS1 e SLC26A4 foram altamente abundantes em precipitados ligados a anti-Ago2 ( Figura 3C ). RT-qPCR mostrou que a expressão de SLC26A4 foi significativamente elevada ao longo do tempo com o tratamento de AngII ( Figura 3D ). Além disso, o aumento da expressão e nível de proteína de SLC26A4 causado pelo tratamento com AngII foram diminuídos quando SLC26A4-AS1 foi silenciado ( Figura 3E-3F ). Em uma palavra, SLC26A4-AS1 interage com SLC26A4 em NMVCs.

Para detectar a função de SLC26A4 na hipertrofia cardíaca induzida por AngII, foram realizados ensaios de perda de função após a verificação da eficiência de sh-SLC26A4#1/2 em NMVCs tratados com AngII ( Figura Suplementar S1A ). A regulação para baixo de SLC26A4 reverteu o aumento da área de superfície de NMVCs tratados com AngII ( Figura Suplementar S1B ) e a regulação para cima dos níveis de biomarcadores hipertróficos estimulados pelo tratamento com AngII ( Figura Suplementar S1C-S1H ). Coletivamente, SLC26A4 promove hipertrofia cardíaca induzida por AngII in vitro.

### SLC26A4-AS1 promove hipertrofia cardíaca induzida por AngII via regulação para cima de SLC26A4

**Figura 4:**
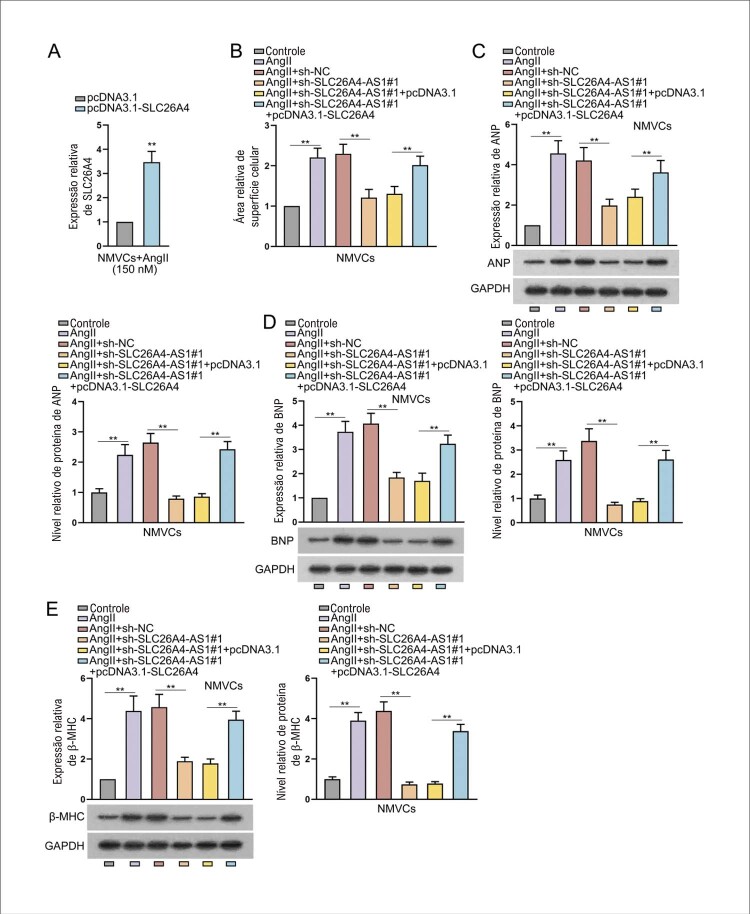
– SLC26A4-AS1 promove hipertrofia cardíaca induzida por AngII via regulação para cima de SLC26A4. (A) NMVCs introduzidos com pcDNA3.1-SLC26A4, ou pcDNA3.1 foram tratados com AngII por 24 h. **P<0,01: pcDNA3.1-SLC26A4 vs pcDNA3.1. Experimentos de resgate foram conduzidos em NMVCs com a transfecção de sh-NC, sh-SLC26A4-AS1#1, sh-SLC26A4-AS1#1+pcDNA3.1 e sh-SLC26A4-AS1#1+pcDNA3.1-SLC26A4 sob tratamento com AngII . n = 3. (B) A área da superfície celular foi avaliada em NMVCs tratados com AngII usando coloração IF. **P<0,01: AngII vs Controle. **P<0,01: AngII+sh-SLC26A4-AS1#1 vs AngII+sh-NC. **P<0,01: AngII+sh-SLC26A4-AS1#1+pcDNA3.1-SLC26A4 vs AngII+sh-SLC26A4-AS1#1+pcDNA3.1. n = 3. (C-E) O nível de expressão e o nível de proteína de biomarcadores hipertróficos em NMVCs tratados com AngII foram medidos por meio de RT-qPCR e western blot, respectivamente, seguidos pela quantificação de western blot. **P<0,01: AngII vs Controle. **P<0,01: AngII+sh-SLC26A4-AS1#1 vs AngII+sh-NC. **P<0,01: AngII+sh-SLC26A4-AS1#1+pcDNA3.1-SLC26A4 vs AngII+sh-SLC26A4-AS1#1+pcDNA3.1. n = 3.

Em seguida, a eficiência de pcDNA3.1-SLC26A4 foi certificada em NMVCs tratados com AngII ( Figura 4A ). Conforme mostrado na Figura 4B-4E , a área de superfície celular e os níveis de biomarcadores hipertróficos em NMVCs tratados com AngII foram obviamente reduzidos com a regulação para baixo de SLC26A4-AS1. No entanto, este efeito de inibição foi revertido após a co-transfecção de pcDNA3.1-SLC26A4. Tomados em conjunto, SLC26A4-AS1 promove hipertrofia cardíaca induzida por AngII através da regulação para cima de SLC26A4.

### SLC26A4-AS1 regula para cima SLC26A4 através do funcionamento como esponjas de miR-301a-3p ou miR-301b-3p

**Figura 5:**
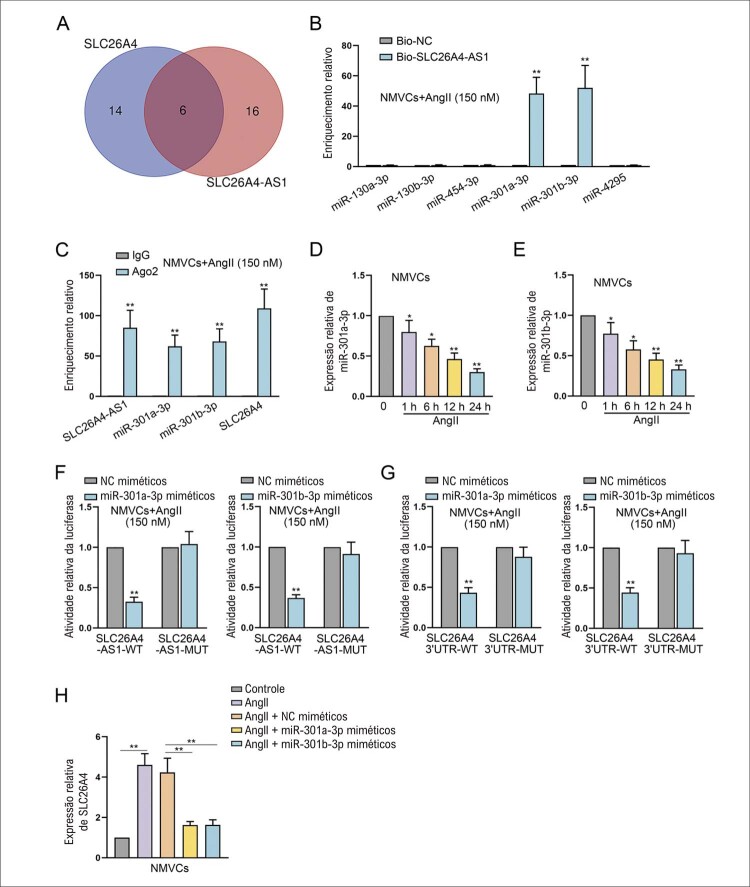
– SLC26A4-AS1 regula para cima a expressão de SLC26A4 por meio de absorção de miR-301a-3p ou miR-301b-3p. (A) Os miRNAs candidatos combinados com SLC26A4-AS1 e SLC26A4 foram obtidos no site da starBase (http://starbase.sysu.edu.cn). (B) O enriquecimento relativo de miRNAs candidatos em grupos bio-SLC26A4-AS1 foi analisado por meio do ensaio de RNA pull-down. **P<0,01: Bio-SLC26A4-AS1 vs Bio-NC. n = 3. (C) O ensaio RIP detectou o enriquecimento relativo de SLC26A4-AS1, miR-301a-3p, miR-301b-3p e SLC26A4. **P<0,01: Ago2 vs IgG. n = 3. (D-E) A expressão de miR-301a-3p ou miR-301b-3p em NMVCs tratados com 150 nM AngII por 1, 6, 12 e 24 h foi detectada usando RT-qPCR. *P<0,05: AngII (1h)/AngII (6h) vs AngII (0 h). **P<0,01: AngII (12h)/AngII (24h) vs AngII (0 h). n = 3. (F) Ensaios de luciferase repórter foram implementados para detectar a atividade de luciferase de SLC26A4-AS1 do tipo selvagem (WT) ou do tipo mutante (MUT). **P<0,01: mímicos miR-301a-3p/mímicos miR-301b-3p vs mímicos NC. n = 3. (G) Ensaios de repórter de luciferase foram realizados para detectar a atividade de luciferase de SLC26A4 3’UTR WT ou MUT. **P<0,01: mímicos miR-301a-3p/mímicos miR-301b-3p vs miméticos NC. n = 3. (H) Os efeitos da superexpressão de miR-301a-3p ou miR-301b-3p na expressão de SLC26A4 foram avaliados via RT-qPCR. **P<0,01: AngII vs Controle. **P<0,01: miméticos AngII+miR-301a-3p/miméticos miR-301b-3p vs miméticos AngII+NC. n = 3.

É bem reconhecido que os lncRNAs podem funcionar como concorrentes de RNAs endógenos (ceRNAs) para regular a expressão gênica por meio da interação com miRNAs em níveis pós-transcricionais.^18^ Seis miRNAs foram selecionados para determinar os miRNAs alvo combinados com SLC26A4-AS1 e SLC26A4 ( Figura 5A ). MiR-301a-3p e miR-301b-3p foram notavelmente abundantes no grupo bio-SLC26A4-AS1 em relação ao grupo bio-NC ( Figura 5B ). Além disso, SLC26A4-AS1, miR-301a-3p, miR-301b-3p e SLC26A4 foram todos marcadamente enriquecidos no grupo Ago2 ( Figura 5C ), indicando a presença de rede de ceRNA entre esses quatro RNAs. A RT-qPCR revelou que tanto o miR-301a-3p quanto o miR-301b-3p foram pouco expressos em NMVCs tratados com AngII de maneira dependente do tempo ( Figura 5D-5E ). Após a verificação da eficiência da superexpressão de miR-301a-3p ou miR-301b-3p ( Figura Suplementar S2A-S2B ), descobrimos que a área de superfície celular e os níveis de biomarcadores hipertróficos em NMVCs induzidos por AngII foram acentuadamente diminuídos por miR-301a -3p ou superexpressão de miR-301b-3p ( Figura Suplementar S2C-S2F ). Todos esses dados sugeriram que miR-301a-3p ou miR-301b-3p poderiam suprimir a hipertrofia cardíaca induzida por AngII in vitro.

Conforme mostrado na Figura Suplementar S3B , os respectivos locais de ligação dos dois miRNAs em SLC26A4-AS1 foram previstos a partir do banco de dados starBase. MiR-301a-3p ou miR-301b-3p superexpressos reduziram significativamente a atividade de SLC26A4-AS1 de tipo selvagem em vez de SLC26A4-AS1-MUT ( Figura 5F ). As sequências de ligação de miR-301a-3p ou miR-301b-3p em SLC26A4 3’UTR também foram mostradas na Figura Suplementar 3B . A atividade de luciferase de SLC26A4 3’UTR tipo selvagem foi diminuída em NMVCs induzidos por AngII após a superexpressão de miR -301a-3p ou miR-301b-3p ( Figura 5G ). Além disso, verificou-se que miméticos de miR-301a-3p ou miméticos de miR-301b-3p podem prejudicar a expressão de SLC26A4 em NMVCs induzidos por AngII ( Figura 5H ). Resumindo, SLC26A4-AS1 regula para cima a expressão de SLC26A4 servindo como um ceRNA para absorção de miR-301a-3p/miR-301b-3p.

### SLC26A4-AS1 afeta a hipertrofia cardíaca induzida por AngII via absorção de miR-301a-3p ou miR-301b-3p

**Figura 6:**
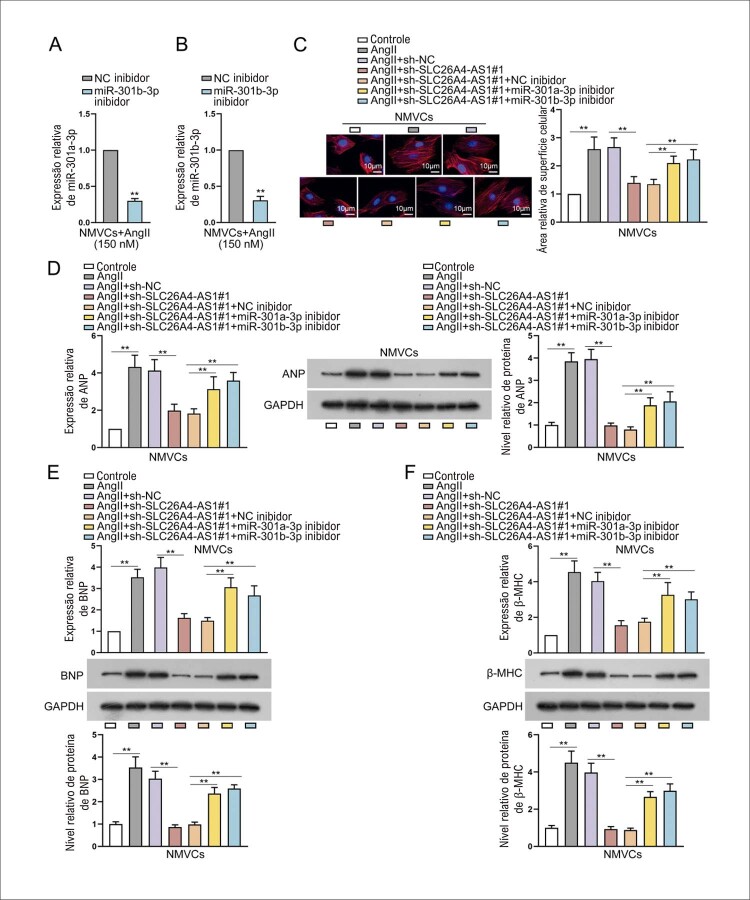
– SLC26A4-AS1 afeta a hipertrofia cardíaca induzida por AngII via absorção de miR-301a-3p ou miR-301b-3p. (A-B) NMVCs tratados com inibidor de miR-301a-3p/inibidor de miR-301b-3p ou inibidor de NC foram tratados com AngII por 24 h. **P<0,01: inibidor de miR-301a-3p/inibidor de miR-301b-3p vs inibidor de NC. Experimentos de resgate foram conduzidos em NMVCs com a transfecção de sh-NC, sh-SLC26A4-AS1#1, sh-SLC26A4-AS1#1+inibidor de NC, sh-SLC26A4-AS1#1+miR-301a-3p inibidor e sh -SLC26A4-AS1#1+miR-301b-3p inibidor sob tratamento com AngII. n = 3. (C) A área da superfície celular foi medida em NMVCs tratados com AngII usando coloração IF. Barra de escala: 10 μm. **P<0,01: AngII vs Controle. **P<0,01: AngII+sh-SLC26A4-AS1#1 vs AngII+sh-NC. *P<0,05: AngII+sh-SLC26A4-AS1#1+inibidor de miR-301a-3p/inibidor de miR-301b-3p vs AngII+sh-SLC26A4-AS1#1+inibidor de NC. n = 3. (D-F) RT-qPCR e western blot avaliaram o nível de expressão e o nível de proteína de biomarcadores hipertróficos em NMVCs tratados com AngII, respectivamente, seguido pela quantificação de western blot. **P<0,01: AngII vs Controle. **P<0,01: AngII+sh-SLC26A4-AS1#1 vs AngII+sh-NC. *P<0,05: AngII+sh-SLC26A4-AS1#1+inibidor de miR-301a-3p/inibidor de miR-301b-3p vs AngII+sh-SLC26A4-AS1#1+inibidor de NC. n = 3.

Para explorar se SLC26A4-AS1 afeta a hipertrofia cardíaca induzida por AngII por meio da regulação de miR-301a-3p/miR-301b-3p, primeiro inibimos a expressão de miR-301a-3p e miR-301b-3p em NMVCs induzidos por AngII ( Figura 6A-6B ). A área de superfície celular diminuída causada pelo silenciamento de SLC26A4-AS1 foi revertida após a cotransfecção do inibidor de miR-301a-3p ou do inibidor de miR-301b-3p ( Figura 6C ). Da mesma forma, a expressão inibida e o nível de proteína de biomarcadores hipertróficos induzidos pelo silenciamento de SLC26A4-AS1 foram neutralizados quando miR-301a-3p ou miR-301b-3p foram silenciados juntos ( Figura 6D-6F ). Tomados em conjunto, SLC26A4-AS1 afeta a hipertrofia cardíaca induzida por AngII por meio da absorção de miR-301a-3p ou miR-301b-3p in vitro.

## Discussão

A hipertrofia cardíaca é dividida em hipertrofia fisiológica e patológica. A hipertrofia cardíaca patológica é o principal fator de indução para a progressão da insuficiência cardíaca com expressões anormais de genes cardíacos, morfologia cardíaca alterada e remodelamento cardíaco mal-adaptativo.^19^ Nos últimos anos, um grande número de pesquisas revelou que os lncRNAs são reguladores importantes na progressão de hipertrofia cardíaca patológica. O LncRNA PEG10 é regulado para cima em cardiomiócitos primários tratados com fenilefrina e exacerba a hipertrofia cardíaca induzida por fenilefrina através da modulação de HOXA9.^20^ O LncRNA TINCR melhora a hipertrofia cardíaca induzida por AngII através da regulação para baixo de CaMKII.^21^ O LncRNA TUG1 é regulado para cima em células H9c2 hipertróficas tratadas com AngII e promove Hipertrofia cardíaca induzida por AngII via interação com miR-29b-3p.^22^ No presente estudo, descobrimos que um novo lncRNA SLC26A4-AS1 está altamente associado à hipertrofia cardíaca^10^ e regulado para cima em cardiomiócitos hipertróficos induzidos por AngII. Além disso, o silenciamento de SLC26A4-AS1 prejudicou a área de superfície celular e o nível de ANP, BNP e β-MHC em cardiomiócitos hipertróficos induzidos por AngII. O SLC26A4-AS1 tem sido associado ao desenvolvimento de vários tipos de câncer, como carcinoma papilífero de tireoide^23^e glioma,^24^ mas seu papel e mecanismo potencial na hipertrofia cardíaca permanecem pouco claros. Nesta pesquisa, verificamos pela primeira vez a promoção de SLC26A4-AS1 na hipertrofia cardíaca induzida por AngII in vitro.

Outra descoberta desta pesquisa foi que SLC26A4-AS1 regula seu gene SLC26A4 próximo para afetar a hipertrofia cardíaca induzida por AngII. Foi relatado que SLC26A4 aumenta a hipertrofia cardíaca.^25^ Consistentemente com essa evidência, esta pesquisa confirmou que silenciar SLC26A4 suprime a hipertrofia cardíaca induzida por AngII. Além disso, a superexpressão de SLC26A4 poderia compensar o efeito supressor da depleção de SLC26A4-AS1 na hipertrofia cardíaca induzida por AngII.

Estudos recentes demonstraram que os lncRNAs podem funcionar pela rede de ceRNA para conduzir a hipertrofia cardíaca, na qual o lncRNA serve como uma esponja de miRNA para regular a expressão de mRNAs. Zhou et al. demonstraram que o lncRNA UCA1 facilita a progressão da hipertrofia cardíaca modulando competitivamente o miR-184 para afetar a expressão de HOXA9.^26^ Li et al. demonstraram que o lncRNA MIAT acelera a hipertrofia cardíaca induzida por AngII por meio da modulação de miR-93 e TLR4.^9^ Wo et al. descobriram que CHRF promove hipertrofia cardíaca induzida por isoproterenol visando miR-93 e Akt3.^27^ O estudo atual também demonstrou que SLC26A4-AS1 regula a expressão de SLC26A4 por meio do funcionamento como ceRNAs como esponja para miRNAs em um nível pós-transcricional. Depois de encontrar os miRNAs comuns de SLC26A4-AS1 e SLC26A4, revelamos que SLC26A4-AS1 regula SLC26A4 por meio do funcionamento como ceRNAs para esponja de miR-301a-3p ou miR-301b-3p.

Estudos anteriores descobriram que os miRNAs exercem funções vitais na hipertrofia cardíaca. O MiR-22 atua como um modulador vital da hipertrofia dos cardiomiócitos e da remodelação cardíaca.^28^ O MiR-625-5p reprime a hipertrofia cardíaca por meio da interação com CaMKII e STAT3.^29^ O MiR-29a facilita a hipertrofia cardíaca pela via PTEN/AKT/mTOR. MiR-301a-3p e miR-301b-3p também foram relatados como implicados em cânceres humanos, como carcinoma hepatocelular,^30^ câncer de pulmão de células não pequenas^31^ e câncer pancreático.^32^ No entanto, o papel supressor de miR-301a -3p e miR-301b-3p na hipertrofia cardíaca induzida por AngII foi comprovado pela primeira vez. Além disso, a inibição de miR-301a-3p ou miR-301b-3p pode reverter os efeitos repressivos do silenciamento de SLC26A4-AS1 na hipertrofia cardíaca induzida por AngII.

## Conclusão

Em conclusão, esta pesquisa achou que SLC26A4-AS1 regula o eixo miR-301a-3p/miR-301b-3p/SLC26A4 para facilitar a hipertrofia cardíaca induzida por AngII, que lança luz sobre um biomarcador promissor para o tratamento da hipertrofia cardíaca. No entanto, o presente estudo foi realizado apenas em experimentos in vitro para investigar o efeito do SLC26A4-AS1 em cardiomiócitos, o que apresenta certas limitações. Para melhorar nossa compreensão do papel específico e do mecanismo subjacente do SLC26A4-AS1 na hipertrofia cardíaca, realizaremos experimentos in vivo e análise clínico-patológica do SLC26A4-AS1 na hipertrofia cardíaca em nossas pesquisas futuras.
