# Supplementary material for: SLC26A4-AS1 Agrava a Hipertrofia Cardíaca Induzida por AngII Aumentando a Expressão de SLC26A4
Source: Arq Bras Cardiol. 2023 Apr 12;120(4):e20210933. [Article in Portuguese] doi: 10.36660/abc.20210933 (PMC10263427; doi:10.36660/abc.20210933)
Supplement: Translation

# Translation: SLC26A4-AS1 Aggravates AngII-induced Cardiac Hypertrophy by Enhancing SLC26A4 Expression

**Authors:** Xiaoliang Han, Chao Li, Qinjiong Ji, Ling Zhang, Xiaofei Xie, Huijuan Shang, Hong Ye

**Affiliations:** 1 China Departamento de Cardiologia, Anhui Provincial Chest Hospital, (Instituto de Controle de Tuberculose de Anhui), Hefei, Anhui – China; 2 China Departamento de Cardiologia, the Second People’s Hospital of Hefei (Hospital Hefei afiliado à Medical University of Anhui), Hefei, Anhui – China

**Keywords:** Cardiomegaly, RNA, RNA, Long Noncoding

## Abstract

**Background:**

It has been reported that solute carrier family 26 members 4 antisense RNA 1 (SLC26A4-AS1) is highly related to cardiac hypertrophy.

**Objective:**

This research aims to investigate the role and specific mechanism of SLC26A4-AS1 in cardiac hypertrophy, providing a novel marker for cardiac hypertrophy treatment.

**Methods:**

Angiotensin II (AngII) was infused into neonatal mouse ventricular cardiomyocytes (NMVCs) to induce cardiac hypertrophy. Gene expression was detected by quantitative real-time PCR (RT-qPCR). Protein levels were evaluated via western blot. Functional assays analyzed the role of SLC26A4-AS1. The mechanism of SLC26A4-AS1 was assessed by RNA-binding protein immunoprecipitation (RIP), RNA pull-down, and luciferase reporter assays. The P value <0.05 was identified as statistical significance. Student’s t-test evaluated the two-group comparison. The difference between different groups was analyzed by one-way analysis of variance (ANOVA).

**Results:**

SLC26A4-AS1 is upregulated in AngII-treated NMVCs and promotes AngII-induced cardiac hypertrophy. SLC26A4-AS1 regulates its nearby gene solute carrier family 26 members 4 (SLC26A4) via functioning as a competing endogenous RNA (ceRNA) to modulate the microRNA (miR)-301a-3p and miR-301b-3p in NMVCs. SLC26A4-AS1 promotes AngII-induced cardiac hypertrophy via upregulating SLC26A4 or sponging miR-301a-3p/miR-301b-3p.

**Conclusion:**

SLC26A4-AS1 aggravates AngII-induced cardiac hypertrophy via sponging miR-301a-3p or miR-301b-3p to enhance SLC26A4 expression.

## Introduction

Cardiac hypertrophy is an adaptive response for enhanced heart afterload to sustain systolic cardiac function at early stages.^1^Despite that, continuous myocardial hypertrophy is a typical heart disease induced by increased heart workload accompanied by maladaptive cardiac remodeling, leading to aortic stenosis and dilated cardiomyopathy.^2 , 3^Oversize thick ventricular walls and cardiomyocytes are the primary patterns of cardiac hypertrophy.^2 , 3^In recent years, cardiac hypertrophy has become an urgent problem that may lead to a rising incidence of heart failure and sudden death.^3^

The outstanding characteristics of pathologic cardiac hypertrophy are the elevation of fetal genes such as brain natriuretic peptide (BNP), atrial natriuretic peptide (ANP), and beta-myosin heavy chain (β-MHC).^4^Recent studies have proved that many factors, including specific growth factors, peptide hormones, and microRNAs (miRNAs), are accountable for cardiac hypertrophy,^5^but the exact molecular mechanism underlying the progression of cardiac hypertrophy remains unclear.

Long non-coding RNAs (lncRNAs) participate in pathological processes via governing gene expression at epigenetic, transcriptional, and post-transcriptional levels.^6^Growing evidence has unveiled the vital roles of lncRNAs in cardiovascular disease development, including cardiac hypertrophy.^7^For instance, SP1-induced lncRNA SNHG14 promotes cardiac hypertrophy via miR-322-5p/miR-384-5p/PCDH17 network in Angiotensin II (AngII)-induced cardiomyocytes.^8^LncRNA MIAT silencing restrains AngII-induced cardiac hypertrophy via miR-93/TLR4 competing endogenous RNA (ceRNA) axis.^9^LncRNA solute carrier family 26 member 4 antisense RNA 1 (SLC26A4-AS1) has been reported as highly associated with cardiac hypertrophy. ^10^ Nonetheless, the effect and regulatory mechanism of SLC26A4-AS1 in cardiac hypertrophy remain vague.

MiRNAs are another type of non-coding RNA with a length of about 22 nucleotides.^11^MiRNAs usually exert cellular functions via targeting downstream messenger RNAs (mRNAs).^12^Numerous miRNAs have been pointed out as essential regulators in the progression of cardiac hypertrophy.^13^For example, miR-17-5p facilitates AngII-induced cardiac hypertrophy via inhibiting Mfn2 expression and activating the PI3K/AKT/mTOR axis to suppress autophagy.^14^MiR-29a relieves isoproterenol hydrochloride-induced cardiac hypertrophy by repressing PPARδ expression.^15^Considering the vital role of miRNAs in cardiac hypertrophy, we tried to investigate the regulatory mechanism of SLC26A4-AS1 by focusing on the miRNAs that interact with it.

In the current study, the effect of SLC26A4-AS1 on the level of BNP, ANP, and β-MHC was explored to determine the function of SLC26A4-AS1 in AngII-induced cardiac hypertrophy. The regulatory mechanism of SLC26A4-AS1 was investigated via the ceRNA axis. Our study might offer a novel insight into the fundamental mechanisms of lncRNA in cardiac hypertrophy, providing a novel target for cardiac hypertrophy treatment.

## Methods

### Cell culture and treatment

Cardiomyocytes were isolated from the neonatal mice. The ethics committee of our Hospital approved animal experiments. To induce hypertrophy of cardiomyocytes, the extracted hearts were split into pieces and treated with 0.25% trypsin (1/400, Beyotime, Shanghai, China), followed by incubation in Dulbecco’s modified Eagle medium/F-12 (500mL, Thermo Fisher Scientific, Rockford, IL, USA) with 10% FBS (1/10, Invitrogen, Carlsbad, CA, USA). After cell confluence reached 80%, cells in the dishes were transferred to culture plates and inoculated with AngII (150 nM, Sigma-Aldrich, St. Louis, MO, USA) with the concentration of 150 nM for 1, 6, 12, and 24 h to stimulate hypertrophic phenotypes of cardiomyocytes, establishing the cell model of cardiac hypertrophy.

### Cell transfection

Short hairpin RNAs (shRNAs) against SLC26A4-AS1 (sh-SLC26A4-AS1#1/2, 3ul/ug, GenePharma Co., Ltd., Shanghai, China), shRNAs against solute carrier family 26 member 4 (SLC26A4) (sh-SLC26A4#1/2, 3ul/ug, GenePharma Co., Ltd.), negative control of shRNA (sh-NC, 3ul/ug, GenePharma Co., Ltd.), pcDNA3.1-SLC26A4 (3ul/ug, GenePharma Co., Ltd.), pcDNA3.1 empty vector (pcDNA3.1, 3ul/ug, GenePharma Co., Ltd.), microRNA (miR)-301a-3p mimics (3ul/ug, GenePharma Co., Ltd.), miR-301b-3p mimics (3ul/ug, GenePharma Co., Ltd.), (NC mimics, 3ul/ug, GenePharma Co., Ltd.), miR-301a-3p inhibitor (3ul/ug, GenePharma Co., Ltd.), miR-301b-3p inhibitor (3ul/ug, GenePharma Co., Ltd.) and negative control of miRNA inhibitor (NC inhibitor, 3ul/ug, GenePharma Co., Ltd.) were all purchased from GenePharma Co., Ltd. (Shanghai, China). All cell transfections were applied using Lipofectamine 3000 (1.5ug/ml, Invitrogen), followed by the treatment of 150 nM AngII for 24 h.

### Quantitative real-time PCR (RT-qPCR)

Total RNA from cells was extracted by TRIzol (1mL, Invitrogen) reagent. The total RNA concentration was determined by measuring the absorption value at 260nm with a microspectrophotometer. The RNA (1μl) was reversely transcribed to compose complementary DNA using M-MLV reverse transcriptase (10000u, Promega, Madison, WI, USA). Afterward, qPCR was conducted with the SYBR Premix Ex Taq™ II kit (Takara, Japan). The expression of miRNA was normalized to U6, and that of lncRNA and mRNA was normalized to glyceraldehyde-3-phosphate dehydrogenase (GAPDH), which was based on the 2^Ct^method. RT-qPCR analysis was performed as described previously.^16^Three independent assays were conducted. The primer sequence for SLC26A4-AS1 and GAPDH is shown in Table 1 .

**Table 1:** – Primer sequence for SLC26A4-AS1 and GAPDH

Genes	Sequence
SLC26A4-AS1	F:TGCTGTTGCTGGAAAGCGAG R: TATTCCTTCCCGCGTGTCCT
GAPDH	F: GACAGTCAGCCGCATCTTCT R:GCGCCCAATACGACCAAATC

*F: forward primer; R: reverse primer.*

### Western blot

RIPA lysis buffer (100mL, Thermo Fisher Scientific) was used to extract total proteins, and protein concentration was verified using a BCA protein assay kit (Abcam, USA). After the treatment of sodium dodecyl sulfate-polyacrylamide gel electrophoresis (SDS-PAGE, 1/10, Invitrogen), proteins were transferred onto PVDF membrane (Millipore, USA) and blocked in 5% defatted milk. Subsequently, the membrane was cultured with primary antibodies against ANP (1µg/ml, ab237632, Abcam, Cambridge, MA, USA), BNP (1µg/ml, ab92500, Abcam), β-MHC (1/2500, ab55152, Abcam), SLC26A4 (0.25 µg/μl, 20889-1-AP, Proteintech, Chicago, USA) and GAPDH (1/2500, ab8245, Abcam) at 4°C overnight. After being washed, the membrane was cultured with HRP-conjugated secondary antibody (1/2500, Abcam) for 1 h. The signal was measured using the enhanced chemiluminescence western blotting substrate. All independent experiments were repeated three times.

### Immunofluorescence (IF) staining and cell surface area assay

Cells were washed and fixed and were blocked with normal goat serum containing 1% BSA (1/100, Sigma-Aldrich). Subsequently, cells were cultured with anti-α-actinin (1/100, Sigma-Aldrich) at 4°C for a whole night and were further hatched with a secondary antibody for 1 h. DAPI (4’,6-diamidino-2-phenylindole, 1/2000, Abcam) was applied to stain the nuclei, and a fluorescence microscope was used to capture the images and assess the cell surface area. Three independent assays were conducted.

### Subcellular fractionation

A PARIS kit (Thermo Scientific, USA) was applied for isolating the cytoplasmic and nuclear fractions from neonatal mouse ventricular cardiomyocytes (NMVCs). GAPDH, U6, or SLC26A4-AS1 expression in the nucleus and cytoplasm was assessed by RT-qPCR analysis. Three independent assays were conducted.

### Fluorescent in situ hybridization (FISH)

Alexa Fluor 555-labeled SLC26A4-AS1 probes (50ng, RiboBio, Guangzhou, China) were synthesized by RiboBio (Guangzhou, China). FISH experiments were implemented with a FISH kit (RiboBio). Cells were seeded into slides for 24 h of incubation. After fixation and permeabilization, cells were cultured with SLC26A4-AS1 probes at 37°C overnight. DAPI was utilized to stain cell nuclei, and the images were observed under a fluorescence microscope. Bio-repeats were conducted in triplicate.

### RNA binding protein immunoprecipitation (RIP)

Based on the instructions, the Magna RIP RNA-binding protein immunoprecipitation kit (Millipore) and the Ago2 antibody (1/30, Abcam) were used to perform RIP assays. Co-precipitated RNAs were analyzed via RT-qPCR. Three independent assays were conducted.

### RNA pull-down assay

NMVCs were transfected with a biotinylated SLC26A4-AS1 probe for 48 h. Then collected cells were cultured with M-280 streptavidin magnetic beads (10 mg/ml, Invitrogen). The bound RNAs were assessed via RT-qPCR. Three independent assays were conducted.

### Luciferase reporter assay

The sequence of SLC26A4-AS1 or SLC26A4 3’UTR containing the predicted binding site with miR-301a-3p and miR-301b-3p were severally cloned into the pmirGLO dual luciferase vector (1 µg, Promega), as well as the putative and mutated sequences. Cells (3 × 10^4^/well) were grown in 24-well plates for incubation. Cells were then transfected with these reporter plasmids and miR-301a-3p mimics or miR-301b-3p mimics, respectively. After 48 h transfection using Lipofectamine 3000 reagent, a Dual-Luciferase Reporter Assay kit (Promega) was applied to measure the relative luciferase activity. Three independent assays were conducted.

### Statistical analysis

All experiments were in triplicate, and all independent experiments were repeated three times. The sample size in this study was defined using the random sampling method and calculated using the formula n=2σ2/δ2 f(α, β), where δ is the minimum difference, σ is the total standard deviation, α is inspection level (=0.05), and β is the rate of type II mistake (=0.10). Data were shown as the mean ± standard deviation for 3 independent assays. GraphPad Prism Version 5.0 Software was applied to analyze data. Data normality was checked using the Shapiro-Wilk test, which showed that all data followed the normal distribution. The statistical significance among groups was investigated by the unpaired Student’s t-test or one-way analysis of variance, followed by a post hoc test (Tukey and Dunnett). Besides, a p value less than 0.05 was identified as statistically significant.

## Results

### SLC26A4-AS1 is upregulated in cardiomyocyte hypertrophy induced by AngII

To evaluate the specific role of SLC26A4-AS1 in cardiac hypertrophy, we first treated NMVCs with AngII to induce hypertrophic phenotypes of cardiomyocytes. The levels of hypertrophic biomarkers (ANP, BNP, and β-MHC) in AngII-treated NMVCs were gradually increased over time ( Figure 1A-1C ). Staining showed that the surface area of AngII-treated NMVCs was gradually increased over time ( Figure 1D ). Besides, significant up-regulation of SLC26A4-AS1 in NMVCs was also observed as time-dependent after AngII treatment ( Figure 1E ). In a word, SLC26A4-AS1 is upregulated in AngII-induced hypertrophy of cardiomyocytes.

**Figure 1:** – SLC26A4-AS1 is upregulated in AngII-induced cardiac hypertrophy. (A-C) In NMVCs treated with 150 nM AngII for 1, 6, 12, and 24 h, the expression of hypertrophic biomarkers (ANP, BNP, and β-MHC) was assessed using RT-qPCR, and the protein level of these biomarkers was evaluated through western blot, followed by western blot quantification. *P<0.05: AngII (1 h) vs AngII (0 h). *P<0.05: AngII (6 h) vs AngII (0 h). **P<0.01: AngII (12 h) vs AngII (0 h). **P<0.01: AngII (24 h) vs AngII (0 h). The number of sample size (n) = 3. (D) The cell surface area in NMVCs treated with 150 nM AngII for 1, 6, 12, and 24 h was assessed using IF staining. Scale bar: 10 μm. *P<0.05: AngII (1 h) vs AngII (0 h). *P<0.05: AngII (6 h) vs AngII (0 h). **P<0.01: AngII (12 h) vs AngII (0 h). **P<0.01: AngII (24 h) vs AngII (0 h). n = 3. (E) SLC26A4-AS1 expression in NMVCs treated with 150 nM AngII for 1, 6, 12, and 24 h was detected using RT-qPCR. *P<0.05: AngII (1 h) vs AngII (0 h). *P<0.05: AngII (6 h) vs AngII (0 h). **P<0.01: AngII (12 h) vs AngII (0 h). **P<0.01: AngII (24 h) vs AngII (0 h). n = 3.

### SLC26A4-AS1 facilitates cardiac hypertrophy which was induced by AngII

Furthermore, RT-qPCR analysis verified the successful transfection of sh-SLC26A4-AS1#1/2 plasmids in AngII-induced NMVCs ( Figure 2A ). The increased cell surface area in NMVCs caused by AngII was attenuated by SLC26A4-AS1 silencing ( Figure 2B ). Meanwhile, the upregulated expression and protein level of ANP, BNP, and β-MHC induced by AngII were reversed after SLC26A4-AS1 knockdown ( Figure 2C-2H ). These results uncovered that SLC26A4-AS1 promotes AngII-induced cardiac hypertrophy in vitro.

**Figure 2:** – SLC26A4-AS1 silence suppresses AngII-induced cardiac hypertrophy. (A) NMVCs were treated with sh-SLC26A4-AS1#1/2 or sh-NC after the treatment of AngII for 24 h. **P<0.01: AngII vs Control. **P<0.01: AngII+sh-SLC26A4-AS1#1/2 vs AngII+sh-NC. n = 3. (B) Cell surface area was detected in AngII-treated NMVCs upon SLC26A4-AS1 silence using IF staining. Scale bar: 10 μm. **P<0.01: AngII vs Control. **P<0.01: AngII+sh-SLC26A4-AS1#1/2 vs AngII+sh-NC. n = 3. (C-H) After SLC26A4-AS1 silencing, the expression of hypertrophic biomarkers in AngII-treated NMVCs was measured using RT-qPCR, and the level of these biomarkers was assessed using western blot, followed by the quantification of western blot. **P<0.01: AngII vs Control. **P<0.01: AngII+sh-SLC26A4-AS1#1/2 vs AngII+sh-NC. n = 3.

### SLC26A4-AS1 interacts with SLC26A4, and SLC26A4 promotes cardiac hypertrophy induced by AngII

Subcellular fractionation and FISH assays were first implemented to study the potential mechanism of SLC26A4-AS1 in AngII-induced cardiac hypertrophy. We uncovered that SLC26A4-AS1 was preferentially located in the cell cytoplasm, suggesting the post-transcriptional role of SLC26A4-AS1 in AngII-treated NMVCs ( Figure 3A-3B ). It has been reported that lncRNAs can regulate their nearby genes to exert functions in developing diseases.^17^As shown in Figure Supplementary S3A , the UCSC database (http://genome.ucsc.edu/) predicted that SLC26A4 was a nearby gene of SLC26A4-AS1. Therefore, we conjectured that SLC26A4-AS1 might regulate SLC26A4 to affect AngII-induced cardiac hypertrophy. Then, Ago2-RIP assays were performed for that Ago2 is the central component of RNA-induced silencing complex^22^. SLC26A4-AS1 and SLC26A4 were highly abundant in anti-Ago2 bound precipitates ( Figure 3C ). RT-qPCR displayed that SLC26A4 expression was significantly elevated over time with the treatment of AngII ( Figure 3D ). Besides, the increased expression and protein level of SLC26A4 caused by AngII treatment were diminished when SLC26A4-AS1 was silenced ( Figure 3E-3F ). In a word, SLC26A4-AS1 interacts with SLC26A4 in NMVCs.

**Figure 3:** – SLC26A4-AS1 functions as a ceRNA to regulate SLC26A4 in cardiac hypertrophy induced by AngII. (A-B) Subcellular fractionation and FISH assays were performed to determine the location of SLC26A4-AS1 in AngII-induced NMVCs. n = 3. (C) RIP assays detected the enrichment of SLC26A4-AS1 and SLC26A4. **P<0.01: Ago2 vs IgG. n = 3. (D) The expression of SLC26A4 in NMVCs treated with 150 nM AngII for 1, 6, 12, and 24 h was assessed using RT-qPCR. *P<0.05: AngII (1 h) vs AngII (0 h). *P<0.05: AngII (6 h) vs AngII (0 h). **P<0.01: AngII (12 h) vs AngII (0 h). **P<0.01: AngII (24 h) vs AngII (0 h). n = 3. (E-F) Expression level and protein level of SLC26A4 in AngII-treated NMVCs were assessed using RT-qPCR and western blot, respectively, after SLC26A4-AS1 down-regulation, followed by the quantification of western blot. **P<0.01: AngII vs Control. **P<0.01: AngII+sh-SLC26A4-AS1#1/2 vs AngII+sh-NC. n = 3.

To detect the function of SLC26A4 in AngII-induced cardiac hypertrophy, loss-of-function assays were performed after the efficiency of sh-SLC26A4#1/2 in AngII-treated NMVCs was verified ( Figure Supplementary S1A ). SLC26A4 downregulation reversed the increased surface area of AngII-treated NMVCs ( Figure Supplementary S1B ) and the upregulation of hypertrophic biomarker levels stimulated by AngII treatment ( Figure Supplementary S1C-S1H ). Collectively, SLC26A4 promotes AngII-induced cardiac hypertrophy in vitro.

### SLC26A4-AS1 promotes AngII-induced cardiac hypertrophy via upregulating SLC26A4

Then, the efficiency of pcDNA3.1-SLC26A4 was certified in AngII-treated NMVCs ( Figure 4A ). As shown in Figure 4B-4E , cell surface area and the levels of hypertrophic biomarkers in AngII-treated NMVCs were obviously reduced with SLC26A4-AS1 downregulation. However, this inhibition effect was reversed after co-transfection of pcDNA3.1-SLC26A4. Taken together, SLC26A4-AS1 promotes AngII-induced cardiac hypertrophy through upregulating SLC26A4.

**Figure 4:** – SLC26A4-AS1 promove hipertrofia cardíaca induzida por AngII via regulação para cima de SLC26A4. (A) NMVCs introduzidos com pcDNA3.1-SLC26A4, ou pcDNA3.1 foram tratados com AngII por 24 h. **P<0,01: pcDNA3.1-SLC26A4 vs pcDNA3.1. Experimentos de resgate foram conduzidos em NMVCs com a transfecção de sh-NC, sh-SLC26A4-AS1#1, sh-SLC26A4-AS1#1+pcDNA3.1 e sh-SLC26A4-AS1#1+pcDNA3.1-SLC26A4 sob tratamento com AngII . n = 3. (B) A área da superfície celular foi avaliada em NMVCs tratados com AngII usando coloração IF. **P<0,01: AngII vs Controle. **P<0,01: AngII+sh-SLC26A4-AS1#1 vs AngII+sh-NC. **P<0,01: AngII+sh-SLC26A4-AS1#1+pcDNA3.1-SLC26A4 vs AngII+sh-SLC26A4-AS1#1+pcDNA3.1. n = 3. (C-E) O nível de expressão e o nível de proteína de biomarcadores hipertróficos em NMVCs tratados com AngII foram medidos por meio de RT-qPCR e western blot, respectivamente, seguidos pela quantificação de western blot. **P<0,01: AngII vs Controle. **P<0,01: AngII+sh-SLC26A4-AS1#1 vs AngII+sh-NC. **P<0,01: AngII+sh-SLC26A4-AS1#1+pcDNA3.1-SLC26A4 vs AngII+sh-SLC26A4-AS1#1+pcDNA3.1. n = 3.

### SLC26A4-AS1 positively regulates SLC26A4 via functioning as sponges of miR-301a-3p or miR-301b-3p

It is well-acknowledged that lncRNAs can function as competing for endogenous RNAs (ceRNAs) to regulate gene expression via interacting with miRNAs at post-transcriptional levels.^18^Six miRNAs were sifted out to determine the target miRNAs combined with SLC26A4-AS1 and SLC26A4 ( Figure 5A ). MiR-301a-3p and miR-301b-3p were notably abundant in the bio-SLC26A4-AS1 group relative to the bio-NC group ( Figure 5B ). Furthermore, SLC26A4-AS1, miR-301a-3p, miR-301b-3p, and SLC26A4 were all markedly enriched in the Ago2 group ( Figure 5C ), indicating the presence of ceRNA network among these four RNAs. RT-qPCR revealed that both miR-301a-3p and miR-301b-3p were lowly expressed in AngII-treated NMVCs in a time-dependent manner ( Figure 5D-5E ). After the efficiency of the overexpression of miR-301a-3p or miR-301b-3p was verified Figure Supplementary S2A-S2B ), we found that the cell surface area and hypertrophic biomarker levels in AngII-induced NMVCs were markedly decreased by miR-301a-3p or miR-301b-3p overexpression ( Figure Supplementary S2C-S2F ). All these data suggested that miR-301a-3p or miR-301b-3p could suppress AngII-induced cardiac hypertrophy in vitro.

**Figure 5:** – SLC26A4-AS1 positively regulates SLC26A4 expression via sponging miR-301a-3p or miR-301b-3p. (A) Candidate miRNAs combined with SLC26A4-AS1 and SLC26A4 were obtained from the starBase website (http://starbase.sysu.edu.cn). (B) The relative enrichment of candidate miRNAs in bio-SLC26A4-AS1 groups was analyzed via RNApull-down assay. **P<0.01: Bio-SLC26A4-AS1 vs Bio-NC. n = 3. (C) The RIP assay detected the relative enrichment of SLC26A4-AS1, miR-301a-3p, miR-301b-3p, and SLC26A4. **P<0.01: Ago2 vs IgG. n = 3. (D-E) Expression of miR-301a-3p or miR-301b-3p in NMVCs treated with 150 nM AngII for 1, 6, 12, and 24 h was detected using RT-qPCR. *P<0.05: AngII (1h)/AngII (6h) vs AngII (0 h). **P<0.01: AngII (12h)/AngII (24h) vs AngII (0 h). n = 3. (F) Luciferase reporter assays were implemented to detect the luciferase activity of SLC26A4-AS1 wild-type (WT) or mutant-type (MUT). **P<0.01: miR-301a-3p mimics/miR-301b-3p mimics vs NC mimics. n = 3. (G) Luciferase reporter assays were performed to detect the luciferase activity of SLC26A4 3’UTR WT or MUT. **P<0.01: miR-301a-3p mimics/miR-301b-3p mimics vs NC mimics. n = 3. (H) The effects of miR-301a-3p or miR-301b-3p overexpression on SLC26A4 expression were assessed via RT-qPCR. **P<0.01: AngII vs Control. **P<0.01: AngII+miR-301a-3p mimics/miR-301b-3p mimics vs AngII+NC mimics. n = 3.

As shown in Figure Supplementary S3B , the respective binding sites of the two miRNAs on SLC26A4-AS1 were predicted from the starBase database. Overexpressed miR-301a-3p or miR-301b-3p significantly reduced the activity of wild-type SLC26A4-AS1 rather than that of SLC26A4-AS1-MUT ( Figure 5F ). The binding sequences of miR-301a-3p or miR-301b-3p on SLC26A4 3’UTR were also shown in Figure Supplementary S3B . The luciferase activity of SLC26A4 3’UTR wild-type was decreased in AngII-induced NMVCs after the overexpression of miR-301a-3p or miR-301b-3p ( Figure 5G ). Additionally, it turned out that miR-301a-3p mimics or miR-301b-3p mimics could impair the expression of SLC26A4 in AngII-induced NMVCs ( Figure 5H ). To sum up, SLC26A4-AS1 positively regulates SLC26A4 expression via serving as a ceRNA to sponge miR-301a-3p/miR-301b-3p.

### SLC26A4-AS1 affects AngII-induced cardiac hypertrophy via sponging miR-301a-3p or miR-301b-3p

To explore whether SLC26A4-AS1 affects AngII-induced cardiac hypertrophy via regulating miR-301a-3p/miR-301b-3p, we firstly inhibited the expression of miR-301a-3p and miR-301b-3p in AngII-induced NMVCs ( Figure 6A-6B ). The declined cell surface area caused by SLC26A4-AS1 silence was reversed after the co-transfection of miR-301a-3p inhibitor or miR-301b-3p inhibitor ( Figure 6C ). Likewise, the inhibited expression and protein level of hypertrophic biomarkers induced by SLC26A4-AS1 knockdown were counteracted when miR-301a-3p or miR-301b-3p were silenced together ( Figure 6D-6F ). Taken together, SLC26A4-AS1 affects AngII-induced cardiac hypertrophy via sponging miR-301a-3p or miR-301b-3p in vitro.

**Figure 6:** – SLC26A4-AS1 affects AngII-induced cardiac hypertrophy via sponging miR-301a-3p or miR-301b-3p. (A-B) NMVCs introduced with miR-301a-3p inhibitor/miR-301b-3p inhibitor, or NC inhibitor were treated with AngII for 24 h. **P<0.01: miR-301a-3p inhibitor/miR-301b-3p inhibitor vs NC inhibitor. Rescue experiments were conducted in NMVCs with the transfection of sh-NC, sh-SLC26A4-AS1#1, sh-SLC26A4-AS1#1+NC inhibitor, sh-SLC26A4-AS1#1+miR-301a-3p inhibitor, and sh-SLC26A4-AS1#1+miR-301b-3p inhibitor under AngII treatment. n = 3. (C) Cell surface area was measured in AngII-treated NMVCs using IF staining. Scale bar: 10 μm. **P<0.01: AngII vs Control. **P<0.01: AngII+sh-SLC26A4-AS1#1 vs AngII+sh-NC. *P<0.05: AngII+sh-SLC26A4-AS1#1+miR-301a-3p inhibitor/miR-301b-3p inhibitor vs AngII+sh-SLC26A4-AS1#1+NC inhibitor. n = 3. (D-F) RT-qPCR and western blot evaluated the expression level and protein level of hypertrophic biomarkers in AngII-treated NMVCs, respectively, followed by the quantification of western blot. **P<0.01: AngII vs Control. **P<0.01: AngII+sh-SLC26A4-AS1#1 vs AngII+sh-NC. *P<0.05: AngII+sh-SLC26A4-AS1#1+miR-301a-3p inhibitor/miR-301b-3p inhibitor vs AngII+sh-SLC26A4-AS1#1+NC inhibitor. n = 3.

## Discussion

Cardiac hypertrophy is divided into physiological and pathological hypertrophy. Pathological cardiac hypertrophy is the main induction factor for the progression of heart failure with abnormal expressions of cardiac genes, altered cardiac morphology, and maladaptive cardiac remodeling.^19^In recent years, many researchers have revealed that lncRNAs are important regulators in the progression of pathological cardiac hypertrophy. LncRNA PEG10 is upregulated in phenylephrine-treated primary cardiomyocytes and exacerbates phenylephrine-induced cardiac hypertrophy through modulating HOXA9.^20^LncRNA TINCR relieves AngII-induced cardiac hypertrophy via down-regulating CaMKII.^21^LncRNA TUG1 is upregulated in AngII-treated hypertrophic H9c2 cells and promotes AngII-induced cardiac hypertrophy via interacting with miR-29b-3p.^22^In the present study, we found that a novel lncRNA SLC26A4-AS1 is highly associated with cardiac hypertrophy^10^and upregulated in AngII-induced hypertrophic cardiomyocytes. Furthermore, SLC26A4-AS1 silencing impaired cell surface area and the level of ANP, BNP, and β-MHC in AngII-induced hypertrophic cardiomyocytes. SLC26A4-AS1 has been associated with the development of several cancers, such as papillary thyroid carcinoma^23^and glioma,^24^but its role and potential mechanism in cardiac hypertrophy remain largely unclear. In this research, we verified the promotion of SLC26A4-AS1 on AngII-induced cardiac hypertrophy in vitro for the first time.

Another finding of this research was that SLC26A4-AS1 regulates its nearby gene SLC26A4 to affect AngII-induced cardiac hypertrophy. SLC26A4 has been reported to increase cardiac hypertrophy.^25^Consistent with this evidence, this research confirmed that silencing SLC26A4 suppresses AngII-induced cardiac hypertrophy. Moreover, SLC26A4 overexpression could offset the suppressive effect of SLC26A4-AS1 depletion on AngII-induced cardiac hypertrophy.

Recent studies have demonstrated that lncRNAs could work by ceRNA network to drive cardiac hypertrophy, in which lncRNA serves as a miRNA sponge to regulate the expression of mRNAs. Zhou et al. have proved that lncRNA UCA1 facilitates cardiac hypertrophy progression by competitively modulating miR-184 to affect HOXA9 expression.^26^Li et al. have demonstrated that lncRNA MIAT accelerates AngII-induced cardiac hypertrophy through modulating miR-93 and TLR4.^9^Wo et al. have uncovered that CHRF promotes isoproterenol-induced cardiac hypertrophy by targeting miR-93 and Akt3.^27^The current study also demonstrated that SLC26A4-AS1 regulates SLC26A4 expression via functioning as ceRNAs to sponge miRNAs at a post-transcriptional level. After finding the common miRNAs of SLC26A4-AS1 and SLC26A4, we revealed that SLC26A4-AS1 regulates SLC26A4 via functioning as ceRNAs to sponge miR-301a-3p or miR-301b-3p.

Previous studies have uncovered that miRNAs exert vital functions in cardiac hypertrophy. MiR-22 acts as a vital modulator of cardiomyocyte hypertrophy and cardiac remodeling.^28^MiR-625-5p represses cardiac hypertrophy via interacting with CaMKII and STAT3.^29^MiR-29a facilitates cardiac hypertrophy via the PTEN/AKT/mTOR pathway. MiR-301a-3p and miR-301b-3p have also been reported to be implicated in human cancers, such as hepatocellular carcinoma,^30^non-small cell lung cancer,^31^and pancreatic cancer.^32^Nonetheless, the suppressive role of miR-301a-3p and miR-301b-3p in AngII-induced cardiac hypertrophy was proved for the first time. Additionally, inhibition of miR-301a-3p or miR-301b-3p could reverse the repressive effects of SLC26A4-AS1 silencing on AngII-induced cardiac hypertrophy.

## Conclusion

In conclusion, this research found that SLC26A4-AS1 regulates the miR-301a-3p/miR-301b-3p/SLC26A4 axis to facilitate cardiac hypertrophy induced by AngII, which shed light on a promising biomarker for cardiac hypertrophy treatment.

Nonetheless, the current study was performed only in vitro experiments to investigate the effect of SLC26A4-AS1 in cardiomyocytes, which poses certain limitations. To improve our understanding of the specific role and underlying mechanism of SLC26A4-AS1 in cardiac hypertrophy, we will perform in vivo experiments and clinicopathological analysis of SLC26A4-AS1 in cardiac hypertrophy in our future research.
